# Another message from Intergalactic Bacterial Command

**DOI:** 10.15252/embr.202153388

**Published:** 2021-07-13

**Authors:** Howy Jacobs

**Affiliations:** ^1^ Tampere University Tampere Finland

**Keywords:** Ecology, Microbiology, Virology & Host Pathogen Interaction

## Abstract

Do prokaryotes have anything to teach us about how to handle outbreaks of viral infection?

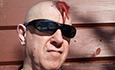

The recent outbreak of SARS‐CoV2 in humans and other animals shows once again that multicellular eukaryotes on planet Earth are especially vulnerable to the effects of viral infection. Do humans have lessons to learn from us, about how to achieve peaceful coexistence with such an enemy?

Indeed, we have many natural strategies for resisting infection by bacteriophages. But most of these are not directly translatable to multicellular organisms, due to the inflexibility of the multicellular condition and the requirement for the cumbersome procedures of sex to reproduce.

Some humans would argue that most of our strategies involve the barbaric practice of sacrificing the many for the few, and result in decreased fitness of the entire population. Yet these arguments fail to take into account the far greater “sacrifices” inherent in the multicellular lifestyle, even without considering the effects of viral infection. Humans are a prime example: the soma, that is, everything except a handful of germ cells, is routinely discarded between generations. The amount of wasted biomass is colossal, compared with the serene equilibrium achieved by unicellular organisms such as ourselves. The human brain, consisting of some 100 billion cells working in harmony, is claimed to be one of the most sophisticated machines to have evolved. Yet it is part of this disposable waste, its entire contents being lost in each cycle (unless transferred in an even more primitive form for long‐term storage on YouTube, PubMed, Wikipedia and so on). But this can hardly compare with the collective consciousness of the 10^31^ or more prokaryotes on Earth, not even considering the rest of us dispersed throughout the cosmos, which have been accumulating knowledge and wisdom for billions of years. Indeed, humans have only very recently figured out that we too can read (Li *et al*, 2021).

The viruses that infect humans are actually more like us, in the sense that they can evolve in real time, negating humans' primitive version of adaptive immunity and their haphazard strategies to strengthen and manipulate it with vaccines and drugs. Our phage receptors evolve almost as rapidly as the viruses that target them, enabling a substantial proportion of individuals to resist infection, or at least escape its lethal consequences. The CRISPR/Cas system, which humans have begun to hijack for their own puny purposes, provides a highly flexible second line of defence. Beyond that, by enticing phages to integrate into our own chromosomes, we provide a much more secure milieu for them to propagate themselves benignly, rather than through the much riskier and largely self‐destructive lytic cycle, which typically ends in an arms race that they cannot win. Animals and plants have also dabbled in such mechanisms, but the outcome is invariably imperfect, verging on disastrous: the virus eventually breaks free of its containment and can then do immense damage to the host, something that happens to bacteriophages only when their host cell is already doomed. Moreover, we have learned to use lysogenic phages as a convenient additional tool in our evolutionary armoury, to transfer survival genes rapidly amongst ourselves.

Intergalactic Bacterial Command (IGBC) is not, as many might imagine, indifferent to the plight of humans suffering from SARS‐CoV2. Indeed, for every human who succumbs to disease, billions of bacteria and even some of our archaeal cousins are destroyed too, because they inhabit the human body as a convenient, if temporary home. Yet we have had only limited success, so far, in transferring our survival skills to our hosts. For example, the main consequence of the endosymbiotic events that created the major eukaryotic organelles seems to have been the ability to accumulate even more disposable biomass of questionable value. By taking control of the machinery of cell death, endosymbiosis did allow our organellar descendants to implement a crude version of the bacterial self‐sacrifice principle. However, viruses easily subvert this machinery, and the eukaryotic nuclear genome has interfered so much in the process that it is now barely recognizable and of very limited use.

If eukaryotic viruses did evolve so as to pose a serious threat to prokaryotic life, we would of course have to take very drastic action, the exact nature of which is best kept confidential, considering that this article is to appear in a eukaryote‐controlled publication. But an interim solution which might make the eradication of eukaryotes or a new round of endosymbiotic invasion unnecessary would be to evolve the machinery to capture viruses such as SARS‐CoV2 ourselves, and then treat them as we would any other bacteriophage.

Humans are so unintelligent that they probably would not even notice, or might ascribe their apparent victory to the brilliance of their own pharmaceutical engineers in coming up with yet another drug that, in reality, has far more limited efficacy than their “data” would appear to claim.

But perhaps we should just allow eukaryotes to destroy themselves. They are, after all, something of a nuisance, even considering only the terrestrial ecosystem. And their search for evidence that we exist beyond planet Earth also poses some dangers to the wider cosmos that we ignore at our peril.
